# Thin-film composite membrane breaking the trade-off between conductivity and selectivity for a flow battery

**DOI:** 10.1038/s41467-019-13704-2

**Published:** 2020-01-07

**Authors:** Qing Dai, Zhiqiang Liu, Ling Huang, Chao Wang, Yuyue Zhao, Qiang Fu, Anmin Zheng, Huamin Zhang, Xianfeng Li

**Affiliations:** 1 Division of Energy Storage, Dalian National Laboratory for Clean Energy (DNL), Dalian Institute of Chemical Physics, Chinese Academy of Sciences, Dalian, 116023 China; 2 State Key Laboratory of Magnetic Resonance and Atomic and Molecular Physics, National Center for Magnetic Resonance in Wuhan, Key Laboratory of Magnetic Resonance in Biological Systems, Wuhan Institute of Physics and Mathematics, Innovation Academy for Precision Measurement Science and Technology, Chinese Academy of Sciences, Wuhan, 430071 China; 3 University of Chinese Academy of Sciences, Beijing, 100049 China; 4 State Key Laboratory of Catalysis, Dalian Institute of Chemical Physics, Chinese Academy of Sciences, Dalian, 116023 China; 5 School of Materials Science and Engineering, Zhengzhou University, Zhengzhou, Henan 450001 China

**Keywords:** Electrochemistry, Energy, Materials chemistry, Batteries

## Abstract

A membrane with both high ion conductivity and selectivity is critical to high power density and low-cost flow batteries, which are of great importance for the wide application of renewable energies. The trade-off between ion selectivity and conductivity is a bottleneck of ion conductive membranes. In this paper, a thin-film composite membrane with ultrathin polyamide selective layer is found to break the trade-off between ion selectivity and conductivity, and dramatically improve the power density of a flow battery. As a result, a vanadium flow battery with a thin-film composite membrane achieves energy efficiency higher than 80% at a current density of 260 mA cm^−2^, which is the highest ever reported to the best of our knowledge. Combining experiments and theoretical calculation, we propose that the high performance is attributed to the proton transfer via Grotthuss mechanism and Vehicle mechanism in sub-1 nm pores of the ultrathin polyamide selective layer.

## Abstract (web summary)

Low-cost flow batteries with high power density are promising for energy storage, but membranes with simultaneously high ion conductivity and selectivity should be developed. Here the authors report a thin-film composite membrane that breaks the trade-off between ion conductivity and selectivity.

## Introduction

Large-scale energy storage is the key technology to solve the issues of intermittency and instability of renewable energies like wind power and solar energy. Flow batteries, benefiting from the flexible design, high safety, and high efficiency are believed to be one of the most promising candidates for large-scale energy storage. Among all, vanadium flow battery (VFB) is one of the most mature technologies, which are at a commercial demonstration stage^1–3^. However, its relatively low power density results in more material consumption for stacks with specific power supplies. Therefore, the improvement in power density is highly important to yield a cost-effective energy storage system. According to the polarization analysis of a VFB, the limiting factor to improve battery power density is the low ion conductivity of a membrane^4^, which plays the role of impeding vanadium ions while conducting protons to form the internal circuit. The coulombic efficiency (CE) of a VFB is highly related to the membrane selectivity, while, the voltage efficiency (VE) of VFB is mainly determined by the ion conductivity (mainly protons) of a membrane.

The power density of current VFB is hindered by the trade-off between ion conductivity and selectivity of membranes since the selectivity of membranes normally decreases with an increase in ion conductivity. Commonly, to achieve a highly conductive ion-exchange membrane, the ion-exchange capacity should be high, which yet results in a high swelling ratio and hence a low ion selectivity^5,6^. Porous membranes are proved to a promising choice since the morphology of porous membranes can be well tuned in different ways^7–10^.

To achieve a porous membrane with high selectivity by traditional phase-inversion method, a denser and thicker skin layer prefers to form. However, a denser supporting layer with less inter-connected pores can also be formed at the same time, which will lead to a low ion conductivity^7,11^. Thin-film composite membranes (TFCMs) combining an ultrathin selective layer with a highly porous substrate can potentially break the trade-off since the selective layer and porous support can be well-tuned separately^12,13^. And previous works have proved that TFCMs demonstrated very promising prospects in water treatment and organic solvent nanofiltration^14–16^. In this paper, a TFCM was fabricated by introducing an ultrathin polyamide selective layer on porous polyethersulfone/sulfonated polyetheretherketone blend (PES/SPEEK) substrate by interfacial polymerization and the TFCM was introduced into flow battery for the first time. According to our calculation and previous supports^17,18^, the ultrathin polyamide selective layer owns very compact cross-linked structures and sub 1 nm pores, which are perfectly in-between the radius of hydrated vanadium ions (V) and protons (H). These sub 1 nm pores endow the ultrathin selective layer with high resistance to hydrated vanadium ions and low resistance to proton transfer, therefore, the TFCM can break the trade-off between the selectivity of V/H and the proton conductivity, and produce a VFB with super high power density.

## Results

### The crumpled and ultrathin selective layer

**Fig. 1:**
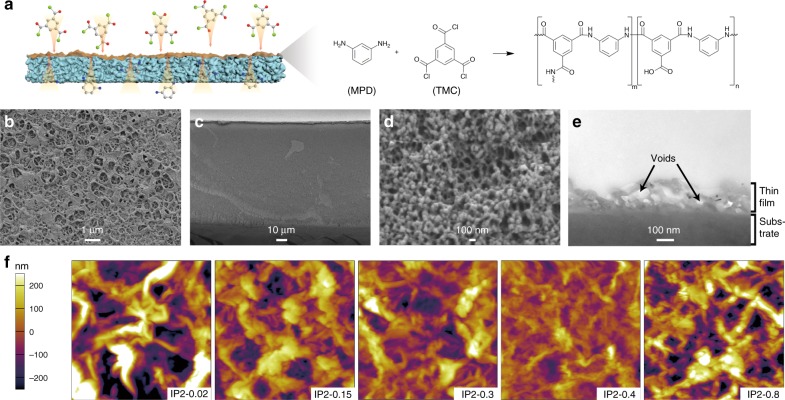
The structure and morphology of thin-film composite membranes and substrate. **a** Schematic illustration of the mechanism of interfacial polymerization. **b** Surface morphology of IP2-0.15. **c**, **d** Cross-section morphology of IP2-0.15. **e** TEM image of the cross-section of IP2-0.15. **f** AFM images of TFCMs. The image size is 5 × 5 μm.

As shown in Fig. 1a, the selective layer was created by interfacial polymerization of trimesoyl chloride (TMC) and m-phenylenediamine (MPD) since the fully aromatic polyamide possesses high stability in acidic condition^19^. The thickness and the morphology were controlled by changing the weight (g)/volume (mL) ratio of TMC from 0.02 to 0.8 wt/v % at a constant MPD concentration of 2 wt/v %. The TFCMs were referred to as IP2-x, where x represents the concentration of TMC. A hydrophilic porous PES/SPEEK substrate (Supplementary Fig. 1) was utilized since the hydrophilic porous structure is beneficial to the formation of an integrated polyamide thin film^16,20^ as well as the ion transfer^5,9^.

The TFCMs possess a “ridge-and-valley-like” selective layer (Fig. 1b and Supplementary Fig. 1) supported on a porous substrate (Fig. 1c, d). Among the five TFCMs, IP2-0.15 possesses the thinnest selective layer (Supplementary Fig. 2). The TEM image of IP2-0.15 (Fig. 1e) shows that the “ridge-and-valley” morphology is created by a crumpled polyamide film with about 40 nm thickness. Voids at the junction of the substrate can be seen under the “ridges” of the polyamide film. As a result, the total thickness of the selective layer is ~180 nm. Though the selective layer is not uniform, the polyamide film is continuous and defect-free. This is benefit from the self-inhibition growth^21,22^ and very fast film formation (within ~10 s)^23,24^. In this process, MPD in the water phase will diffuse through the newly formed polyamide membrane to hexane phase and react with TMC. The diffusion of MPD is driven by concentration gradient and restrained by mass transfer resistance along the MPD diffusion path. Hence, MPD will preferentially diffuse to open areas with low resistance and generate polyamide to block defects, and finally, form a defect-free polyamide film. In addition, the selective layer grows right on the substrate and leaves no continuous gaps between them, resulting in a great intermolecular force to prevent film delamination. The ultrathin polyamide film leads to a very short mass transfer path and the voids separate the selective layer from the substrate and reduce the lateral mass transfer resistance at the interlayer between the selective layer and the substrate^15,16^. Hence, the TFCM is expected to possess a very low resistance. The roughness of TFCMs decreases first and then increases with the increasing TMC concentration (Fig. 1f and Supplementary Fig. 3). This is caused by a competition between a faster consumption of MPD and a higher disturbance caused by a faster reaction when increasing the TMC concentration. The higher the TMC concentration, the faster the MPD will be consumed, the shorter distance the MPD will diffuse for, and the thinner the selective layer will be. However, when the TMC concentration increases further, the faster reaction leads to a faster generation of HCl and heat, resulting in a higher interfacial disturbance that increases the roughness and thickness of the selective layer^14,24^. In short, the crumpled surface increases the area of the selective layer that contacts with electrolytes and is believed to promote mass transfer rate^14^, and thus a TFCM with a thinner and rougher selective layer is expected to possess lower ion resistance.

### Ion selectivity and conductivity

**Fig. 2:**
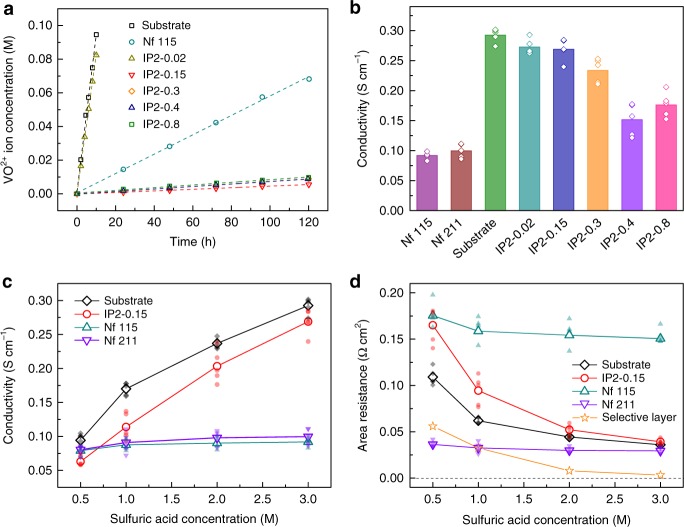
The selectivity and conductivity of thin-film composite membranes. **a** Vanadium concentrations in the magnesium sulfate solution at the permeate side versus time. **b** Cross-membrane conductivity of different membranes. **c**, **d** The change of cross-membrane conductivity (**c**) and area resistance (**d**) with the sulfuric acid concentration. The area resistance of the selective layer equals to the difference between the area resistance of the substrate and of the IP2-0.15. Data behind each average are represented by smaller symbols. Nf represents Nafion. Source data of **b**–**d** are provided as a Source Data file.

A membrane with higher ion selectivity possesses higher resistance to vanadium ions, which is very important in flow batteries. A highly selective membrane can effectively resist reactive substances in opposite electrolytes from contacting with each other and slow down the self discharge. In this work, ion selectivity and conductivity were evaluated by vanadium-permeation experiment and electrochemical impedance spectrum. IP2-0.15 possesses the highest vanadium ion resistance among five TFCMs (Fig. 2a). The difference in vanadium permeance is caused by the matching of reactant concentrations, which has shown great influence on the selectivity of polyamide film^14^. The vanadium ion permeability of IP2-0.15 (4.40 × 10^−6^ cm^2^ h^−1^) calculated by Eq. (3) is two orders of magnitude lower than that of the substrate (8.72 × 10^−4^ cm^2^ h^−1^) and one order lower than does Nafion 115 (7.09 × 10^−5^ cm^2^ h^−1^). This result indicates that the selectivity of these TFCMs are mainly contributed by their selective layer and the polyamide thin film possesses very high resistance to hydrated vanadium ions and is expected to bring the VFBs with very high CE.

The ion conductivities of TFCMs correspond well with the tendency of thickness and roughness of selective layers. IP2-0.15 possesses a higher ion conductivity compared with IP2-0.3, IP2-0.4 (Fig. 2b), due to its thinner selective layer and a relatively higher roughness (Supplementary Figs. 2 and 3). The IP2-0.8 with high roughness shows a lower conductivity, mainly due to its higher thickness. The selective layer of IP2-0.02 is not well-formed, hence its conductivity is the highest among the five TFCMs. To investigate the ion transfer mechanism in polyamide film, membrane ion conductivities in different acid concentrations were measured (Fig. 2c and Supplementary Fig. 5). With increasing acid concentration, the ion conductivity of the substrate and IP2-0.15 increases dramatically, while that of Nafion 115 increases slightly. This is because protons transfer mainly through electrolytes in pores of porous membranes, while protons are trapped by sulfonic groups in Nafion 115^25^. In a 3 M sulfuric acid solution, the ion conductivity of Nafion 115 is about 0.092 S cm^−1^, while that of IP2-0.15 reaches about 0.269 S cm^−1^, which is three times as high as that of Nafion 115. And the area resistance of IP2-0.15 is even comparable with Nafion 211 with a thickness of 25 μm (28 μm after swelling). By subtracting the area resistance of the substrate from IP2-0.15, the estimated area resistance of the selective layer is found to dramatically decrease with the increase of acid concentration (Fig. 2d). As a result, the area resistance of IP2-0.15 is very close to that of the substrate in 3 M sulfuric acid. This result shows that the conductivity of the polyamide selective layer in a higher acid concentration solution is much higher than that in a lower acid concentration solution, indicating an ion transfer behavior in the selective layer other than transfer by the protonation of the amide groups on polymer chains since the ion conductivity will not change dramatically with the acid concentration if protons are trapped in polymer chains.

### The ion transfer mechanisms in polyamide selective layer

**Fig. 3:**
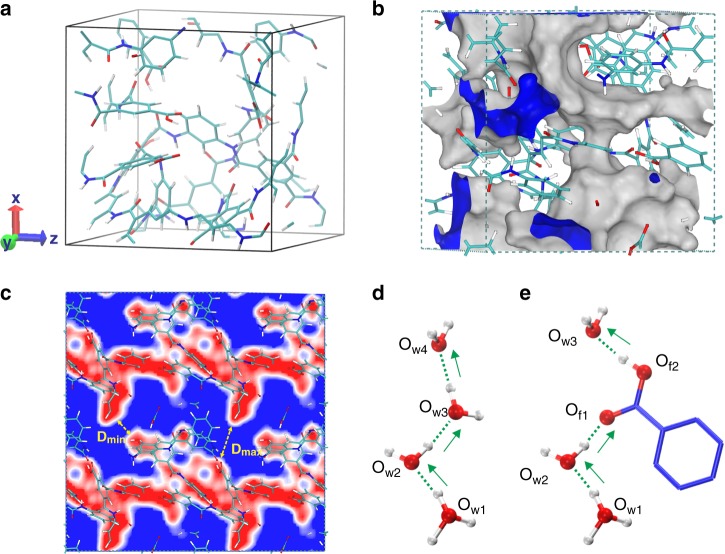
Structure, channel and proton conduction of thin-film composite membranes. **a** The optimized structure. **b** 3D channels (inner surface: blue). **c** 2D channel (accessible area: blue) as well as the framework (unaccessible area: red) parallel to *y* and *z*-axis. The red, blue, cyan and white represents O, N, C and H atom, respectively. **d** Transfer through water molecules. **e** Transfer through carboxyl groups as well as water molecules. Red, blue and white color represents O, C and H atom, respectively.

Theoretical calculation was carried out to further investigate ions transfer mechanisms in polyamide selective layer on the basis of available experimental parameters. The optimized model (Fig. 3a) and channel (Fig. 3b) show that the highly cross-linked and rigid polyamide structure prevents polymer units from compactly stacked. As a result, the cross-linked polyamide framework possesses pores with size distribution (PSD) in a range of 3.6–6.2 Å (diameter (d)) (Supplementary Fig. 6), and the largest free diffusion sphere (*D*_f_), which can diffuse through the pores was calculated to be 3.6 Å. It’s generally accepted that *D*_f_ is considered as a more reliable calibration to estimate the diffusivity because the narrowest part (*D*_min_, while is not *D*_max_ in Fig. 3c) of a continuous irregular channel strongly determines the diffusion behavior and thus the ion selectivity^26,27^. With regards to the size of hydrated vanadium ions (d > 6 Å)^28,29^, the irregular pores will dramatically hinder the mobility of the hydrated vanadium ions but permit the proton to diffuse freely (d~3.0 Å) through the polyamide as illustrated by following advanced ab initio molecular dynamics (AIMD) simulation.

**Fig. 4:**
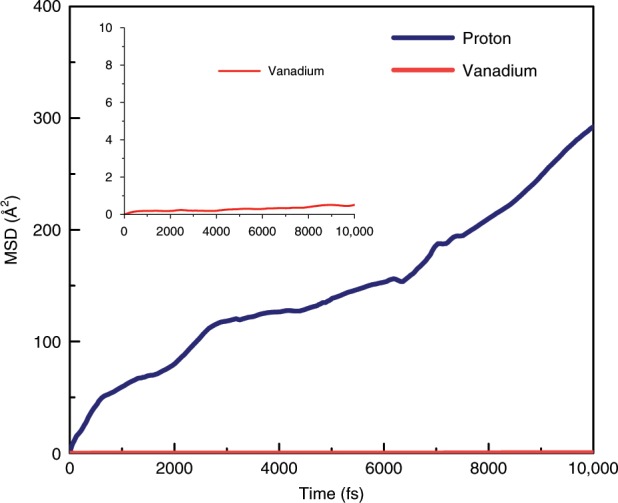
Mean square displacement of proton and vanadium ion in thin-film composite membranes at 298 K. Inserts are enlarged views for vanadium ion.

It is well known that the AIMD simulation is a powerful tool to investigate the proton transport across the membrane^30–32^. In this work, the AIMD was carried out as well to reveal the conductivity mechanism of the TFCMs. As shown in Fig. 3d, the proton could be easily transported exclusively through water. During the process of conduction, the proton was firstly carried out by one water and connected with another water molecule by rotation and translation with vehicle mechanism^33,34^ (Supplementary Fig. 7a). Then the proton hopped into another water molecule and moved through hydrogen-bonding network with Grotthuss mechanism^35–37^ (Supplementary Fig. 7b–d). On the other hand, the carboxyl group (-COOH) played a crucial role in bridging the hydrogen-bonding percolation path between hydronium ions and acidic carboxyl groups, and thus enhance the proton conduction (Fig. 3e). As shown in Supplementary Fig. 8a, the proton was firstly moving as a part of hydronium ion (O_w1_) with vehicle mechanism. Then the proton jumped to another water (O_w2_) with Grotthuss mechanism (Supplementary Fig. 8b). In the next step, the proton diffused into the adjacent carboxyl group (O_f1_) and another proton connected with carboxyl (O_f2_) synchronously hopping into water (O_w3_) molecule with Grotthuss mechanism (Supplementary Fig. 8c, d). This mechanism is in good agreement with the previous work illustrated by Luduena et al^31^. The proton transfer process discussed above is shown in Supplementary Movie 1. Moreover, we also investigated the diffusion of vanadium ions. As shown in Fig. 4, it indicated that vanadium could not diffuse, because the size of hydrated vanadium ions (d > 6 Å) was too large to through the pores of TFCMs. Overall, the long-range proton conduction in TFCMs was principally attributed to Grotthuss mechanism with some synergism with vehicle mechanism involving hydroniums and water as well as acidic carboxyl group, while vanadium ions are highly resisted due to size exclusion.

### Battery performance

**Fig. 5:**
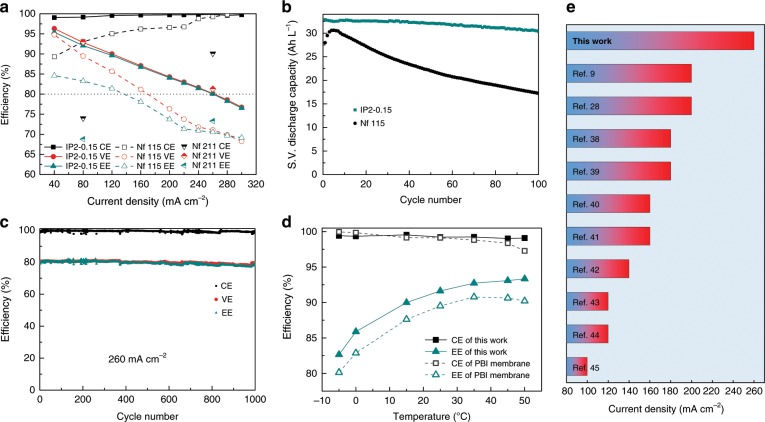
Battery performance. **a** Efficiencies of VFBs equipped IP2-0.15 at different current density compared with VFBs equipped with Nafion 115. **b** Comparison of specific volume (S.V.) discharge capacity change with the cycle number at a current density of 80 mA cm^−2^. **c** Cycle performance of VFBs equipped with IP2-0.15 at a current density of 260 mA cm^−2^. **d** Efficiencies of VFBs equipped with IP2-0.15 and a high-performance porous PBI membrane reported in our previous work^57^. **e** Comparison of the highest current density with an energy efficiency of about 80% reported in recent five years and our work^58–63^.

VFB single cell (Supplementary Fig. 9) tests were carried out on five TFCMs, among which IP2-0.15 performs the best (Supplementary Fig. 10a, b). At a current density of 80 mA cm^−2^, a VFB equipped with IP2-0.15 demonstrated excellent efficiencies with a CE of 99.2%, a VE of 92.8% and an EE of 92.1%, respectively. In contrast, the VFBs equipped with Nafion 115 and the PES/SPEEK porous substrate both show much lower efficiencies (Supplementary Fig. 10c). This result is attributed to the high selectivity and low resistance of IP2-0.15 since the CE increases dramatically from 61.8% of the substrate to 99.2% of IP2-0.15 while the VE does not decline after coating the substrate with an ultrathin polyamide film. At a higher current density, CE increases because of shorter charge-discharge time and VE decreases because of larger polarization (Fig. 5a). Consequently, the energy efficiency decreases. Because of the higher conductivity of IP2-0.15 than that of Nafion 115, the ohmic polarization at high current density is much lower for VFBs equipped with IP2-0.15. Hence, even at a high current density of 260 mA cm^−2^, EE still reaches 80%, which is the highest values ever reported (With very traditional structure: a 5 mm thick carbon felt and effective area of 48 cm^2^, without flow field). As a comparison, a VFB with a Nafion 115 can not be normally charged or discharged in current densities higher than 240 mA cm^−2^ because of its high polarization (Supplementary Fig. 11), as a result, its efficiency is abnormal (Fig. 5a). A VFB with a Nafion 211 can work at a current density of 260 mA cm^−2^, however, its CE is much lower than that of IP2-0.15 membrane.

Moreover, the highly selective IP2-0.15 can effectively resist the electrolyte cross over and demonstrates a much lower rate of capacity decay compared with Nafion 115 (Fig. 5b). As a result, the average capacity decay rate in 100 cycles of IP2-0.15 is −0.07% per cycle while that of Nafion 115 is −0.44% per cycle. To further confirm the reliability of IP2-0.15, VFBs equipped with an IP2-0.15 were operated at a current density of 260 mA cm^−2^ for a long time and tested at different temperatures at 80 mA cm^−2^. The results show that the battery efficiency keeps stable in 1000 cycles (Fig. 5c). The morphology of IP2-0.15 after 1000 cycles show that the selective layer is well reserved but flatter than before (Supplementary Fig. 12). This result demonstrating that the interaction between the selective layer and the substrate is strong enough to prevent the selective layer from delamination. The flatter morphology is caused by the press of electrodes and the hydraulic pressure of electrolyte. The stability of TFCM is further tested by a soaking test (Supplementary Fig. 13). The result shows that the polyamide selective layer remains integrity after soaking in 0.15 M V(V) + 3 M H_2_SO_4_ at 40 °C for 7 days. The concentration of V(IV) in solution as time due to the polymer degradation changed rarely, further confirming the stability of TFCM. The VFB assembled with IP2-0.15 performs much better than those with the outstanding PBI membrane^38^ reported previously at different temperatures (Fig. 5d). Even at the temperature as low as −5 °C, EE of the VFB assembled with IP2-0.15 can reach 82.7%. At 50 °C its CE can still reach 99% and EE can reach 93.3%. In another word, the compact polyamide layer demonstrated super high selectivity, even at high temperatures. Compared with other membranes reported in previous works, the membrane in our work demonstrates the highest current density with an electrode area of 48 cm^2^ and thickness of 5 mm while keeping EE about 80% (Fig. 5e).

## Discussion

In summary, we fabricated a TFCM with an ultrathin selective layer. The ion selectivity is high and the conductivity of the TFCM in 3 M H_2_SO_4_ far exceeds that of Nafion 115. According to our simulation, the cross-linked polyamide selective layer possesses sub-1 nm pores that can well separate hydrated vanadium ions from conducting protons. Vanadium ions are resisted because of size exclusion while protons transfer in the polyamide selective layer is contributed by Grotthuss mechanism and Vehicle mechanism. This thin and compact layer with proper pore size can break the trade-off between ion conductivity and selectivity and achieve a dramatically improved performance for VFB. The vanadium battery assembled with TFCMs can work at the highest current density with energy efficiency higher than 80% ever reported. Since flow batteries are important techniques in energy storage and many new flow batteries bursts out in recent years, the findings from this work can surely boost the development of next-generation high power density flow batteries. The mechanism of proton transfer in the selective layer of TFCM is firstly proposed and the results are expected to guide the research of better TFCMs applied to batteries and filtrations.

## Methods

### Materials

PES was obtained from Changchun Jilin University Special Plastic Engineering Research, with a viscosity of 0.58. Sulfonated poly (ether ether ketone) (SPEEK) was prepared by direct sulfonation of poly (ether ether ketone) (PEEK) with sulfuric acid (98%) at 70 °C for 2 h^39^. MgSO_4_ (AR), H_2_SO_4_ (98%), tetrahydrofuran (AR), and N,N-dimethylacetamide (DMAc, AR) were purchased from Tianjin Damao Chemical Reagent Factory. VOSO_4_ (99%) was purchased from Haizhongtian Chemical Reagent Factory in Shen Yang, China. N-hexane (99.5%) was purchased from Tianjin Kemiou Chemical Reagent Co., Ltd. MPD 99.5% and TMC 98% was purchased from Aladdin, Shanghai, and used as received.

### Preparation of PES/SPEEK porous substrate

PES/SPEEK substrate was prepared by nonsolvent induced phase separation (NIPS) method^40^. PES and SPEEK were dissolved in DMAc to form a homogeneous casting solution with a weight ratio of PES: SPEEK: DMAc = 24:6:70. Then the solution was cast on a glass plate, afterward, the plate was immersed in water to get a porous substrate. The thickness of a doctor blade is 250 μm and the temperature of the water bath is adjusted to 19 ± 1 °C. The thickness of the PES/SPEEK substrate was 105 ± 5 μm and the substrates were stored in water at room temperature.

### Fabrication of TFCMs by interfacial polymerization

2 wt/v % of MPD was dissolved in deionized water to form a water-phase solution and TMC was dissolved in hexane to form the oil-phase solution. At the first step, the PES/SPEEK porous substrate was immersed in the MPD solution for 3 min. At the second step, the substrate was taken out and the water on the water-side surface (skin layer) of the substrate was removed with a tissue paper, and the glass-side surface was coated with a glass sheet. At the third step, the substrate, together with the glass sheet, was immersed into the TMC solution in hexane for 1 min to grow a polyamide thin film on the membrane skin layer. At the last step, the TFCM was taken out and stored in water. Different TFCMs were fabricated by changing the TMC weight to volume percentage in hexane.

### Cross-membrane conductivity

The cross-membrane conductivity was measured by a simple method as follows. One piece of the membrane was sandwiched between two round titanium tablets with a diameter of 1.5 cm and a thickness of 0.3 cm. The surface of titanium tablet was polished to be very smooth and flat. This sandwich was then fixed by a plastic clamp with two pieces of copper sheets at the jaw. The copper sheets were connected to the electrochemical impedance spectroscopy (EIS) testing station (Solartron SI 1260 and SI 1287) to measure the resistance. The frequency range of EIS was set from 1 KHz to 1 MHz and from 1 KHz to 100 kHz alternatively. However, this resistance includes the electric resistance of the device and the contact resistance between the titanium tablet and the membrane. To eliminate the contact resistance and electric resistance of the device, several pieces of membranes were stacked together and a relationship between the resistance and number of layers in membrane stacks was constructed. One membrane sample was divided into 4 pieces and we measured 1 to 4 pieces stacked together and got the resistance of one membrane by calculating the slope of total resistance vs. the number of membranes stacked. An example is provided in Supplementary Fig. 5. The cross-membrane conductivity of one piece of the membrane was calculated according to the following equation.

1 $$\sigma = \frac{T}{{R \times A}}$$

where *σ* (S cm^−1^) is the cross-membrane conductivity. *T* (cm) is the membrane thickness. *R* (Ω) is the resistance of one membrane. And *A* (cm^2^) is the effective area of the membrane which is the area of titanium tablet in this method.

The area resistance can be calculated by the following equation.

2 $$R_{\mathrm{A}} = R \times A$$

All of the membranes were pretreated in 3 M sulfuric acid solution overnight. Before testing the cross-membrane conductivity in different sulfuric acid concentrations, membranes were treated in the corresponding sulfuric acid solution with a specific concentration. We tested the conductivity in 0.5, 1, 2, and 3 M sulfuric acid, respectively. Five samples were tested for each kind of membrane.

### Vanadium ion permeability

The permeability of vanadium ion was tested in a diffusion cell with two 120 mL half cells. VO^2+^ (V(IV)) was chosen as the diffusion ion. One half-cell, the feed side, was filled with 120 mL 1.5 M VOSO_4_ dissolved in 3 M H_2_SO_4_, while another half-cell, the permeation side, was filled with 120 mL 1.5 M MgSO_4_ dissolved in 3 M H_2_SO_4_ to balance the ion strength and reduce the osmotic pressure. Solutions at both sides were kept stirring. 3 mL of the sample solution was collected from the MgSO_4_ side after an equal time and 3 mL of original MgSO_4_ solution was added back. The concentration of V(IV) was determined by UV-vis spectrometer (TU-1901, Beijing Purkinje General Instrument Co., Ltd.). The permeability was calculated by Fick’s diffusion law:

3 $$V_{\mathrm{B}}\frac{{{\mathrm{d}}C_{\mathrm{B}}(t)}}{{{\mathrm{d}}t}} = A\frac{P}{L}\left( {C_{\mathrm{A}} - C_{\mathrm{B}}\left( t \right)} \right)$$

where *V*_B_ is the solution volume on the MgSO_4_ side. *A* is the effective area of the membrane, and *L* is the thickness of the membrane. *P* represents the permeability. *C*_A_ is the ion concentration on the VOSO_4_ side. *C*_B_(*t*) is the ion concentration on the other side.

### Battery performance

A single cell composing of a stainless-steel endplate, carbon current collector, carbon felt (6 cm × 8 cm effective area and 5 mm thick) and membrane in a sandwich configuration was utilized to test the electrochemical performance. The electrolyte volume in each storage tank was 60 mL, and the initial electrolyte composition was 1.5 M V (III)/V (IV) and 3 M H_2_SO_4_. Arbin BT 2000 was used to test the charge-discharge performance. The cut-off voltages were set at 1.55 V and 1.0 V for charge and discharge test to avoid corrosion of electrodes and electrolysis of water. Efficiencies of VFB single cells were evaluated by parameters of CE, VE, and energy efficiency (EE), calculated by following equations. The temperature-dependent efficiencies were tested in a constant temperature chamber (ET-020L-Shanghai Espec).

4 $${\mathrm{CE}} = \frac{{{\mathrm{Discharge}}\,{\mathrm{capacity}}}}{{{\mathrm{Charge}}\,{\mathrm{capacity}}}} \times 100\%$$

5 $${\mathrm{EE}} = \frac{{{\mathrm{Discharge}}\,{\mathrm{energy}}}}{{{\mathrm{Charge}}\,{\mathrm{energy}}}} \times 100\%$$

6 $${\mathrm{VE}} = \frac{{{\mathrm{Average}}\,{\mathrm{discharge}}\,{\mathrm{voltage}}}}{{{\mathrm{Average}}\,{\mathrm{charge}}\,{\mathrm{voltage}}}} \times 100\% = \frac{{{\mathrm{EE}}}}{{{\mathrm{CE}}}} \times 100\%$$

### Measurement of swelling ratio by AFM

A TFCM was put onto a silicon wafer with the selective layer face down. Then, dropwise add DMAc to dissolve the substrate and dry the sample at 120 °C. After drying, the polyamide film sticks to the wafer. DMAc was added again to wash away the residual substrate followed by vacuum drying at 120 °C overnight. Before AFM, the selective layer was scratched with a needle. The polyamide film thickness was measure in contact mode. Dry-state thickness was firstly measured, followed by adding water onto the sample without moving the tip. The thickness was measured repeatedly until the film stops swelling.

The swelling ratio was calculated by:

7 $$S = \frac{{(h_{wet} - h_{dry})}}{{h_{dry}}} \times 100{\mathrm{\% }}$$

where $h_{wet}\,{\mathrm{and}}\,h_{dry}$ are the mean thickness of dry and wet polyamide film, respectively. The result is shown in Supplementary Fig. 14.

### Measurement of density

Several researchers reported on polyamide films’ density based on dry form^14,41^. Since polyamide thin films swell in water, their bulk density in water should be smaller than that in dry form and the channel size should be larger. Therefore, to make our simulation more close to the situation in the electrolyte, the swelling behavior was included in the measurement of density. The true density of polyamide was measured in a 10 mL pycnometer (ASONE, Japan). Polyamide films were prepared by reaction of 50 mL 2% MPD in water and 50 mL 0.15% TMC in n-hexane while stirring for 30 min and filtrated with nonwoven fabrics and washed with water for over five times before being dried in a vacuum oven at 50 °C under −0.1 Mpa overnight. Then, polyamide films were ground into powder in an agate mortar and dried in 100 °C under −0.1 Mpa overnight. When measuring the density, the pycnometer was cleaned and filled with water and record the weight, *m*_1_. Then, the water was poured out and an amount of polyamide powder was added into the pycnometer and the weight of the sample was recorded as the weight increment, *m*_2_. Then, ~2/3 space of the pycnometer was filled with water. At this time, the polyamide powder floated on the water since gas was in powder pores. To remove the gas, the power in pycnometer was repeatedly treated by ultrasonication and vacuumed under −0.06 Mpa at 25 °C, until all of the powder sank to the bottom. Afterward, the rest space of pycnometer was filled with water and weighed as *m*_3_. The density of the polyamide powder was calculated by the equation below:

8 $$\rho _{{\mathrm{true}}} = \frac{{m_2}}{{m_1 + m_2 - m_3}}\rho _{water}$$

The bulk density of polyamide after swelling was calculated by the following equation:

9 $$\rho _{{\mathrm{swelling}}} = \frac{{\rho _{dry}}}{{1 + {\mathrm{S}}}}$$

where $\rho _{dry}$ is the dry-state density of polyamide. We take $\rho _{{\mathrm{true}}}$ as an estimation of $\rho _{dry}$ since dry-state polyamide film is very dense. S is the volume swelling ratio of polyamide, which is measured by AFM.

### Cross-linking degree

The cross-linking degree was calculated by the following equation^42^:

10 $$O/N = \frac{{3m + 4n}}{{3m + 2n}}$$

11 $${\mathrm{{cross}}\,\mathrm {linking}} = \frac{m}{{({\mathrm{m}} + {\mathrm{n}})}}$$

where O/N is the aromic ratio of polyamide. *m* and *n* are shown in Fig. 1a.

### Soaking test

The soaking test was carried out in a 60 mL solution of 0.15 M VO_2_^+^ and 3 M H_2_SO_4_ in 40 °C. Samples were tailored into 5 × 5 cm^2^ squares for test. The oxidization stability is evaluated by the concentration change of VO^2+^ generated from the reduction of VO_2_^+^.

### Characterizations

The surface morphology of membranes was characterized by Field emission scanning electron microscope (FE-SEM, JSM-7800F). To investigate the cross-section morphologies, the membrane samples were cut into strips and broken in liquid nitrogen. The samples were coated with gold before FE-SEM analysis. To determine the thicknesses of polyamide thin films, membranes were encapsulated with epoxy resin (EPON812) and sliced into sub 100 nm slices with a slicer (LEICA EM UC6). Afterward, the slices were carried by copper meshes and investigated by transmission electron microscope (TEM, JEM-2000EX, JEOL). The accelerating voltage is 120 kV and the camera is AMT XR-41. X-ray photoelectron spectrometer (XPS) was adopted to determine the elemental composition of the membrane. XPSPEAK41 was used to fit the data. To correct the shift of binding energy caused by sample charging, the high-resolution C1s peak was fitted and then all binding energy values were shifted until the major peak of C1s was corrected to 285 eV^14^. Atomic force microscope (AFM) was carried out on Cypher from Asylum Research and the probe is NANOWORLD silicon SPM-Sensor with a resonance frequency of 0.7–2.0 MHz.

### Model for polymer framework

Our models were designed by Amorphous Cell module in Materials Studio packages. It is well known that this module is a comprehensive model building tool for creating amorphous materials, which has been extensively used in the previous work.^43–47^ Amorphous Cell constructs the unit cell (or super cell) by growing the polymer chains segment by segment, taking into account interactions with previously positioned segments and the energy of adding the subsequent segment. The rotational isomeric state model and a modified Markov process were used for this purpose. In order to build the desirable model of polyamide which can match with the experimental findings, the model (C_162_H_108_N_24_O_36_) was initially constructed on the basis of experimental density (1.09 g m^−3^), crosslinking degree (0.4) and O/N ratio (1.5) (Supplementary Figs. 4 and 14, Supplementary Tables 1 and 2). During the construction, the Boltzmann distribution, cumulative probability, and degrees of freedom was used to evaluate the accuracy of sampling^48^. Then the Forcite tool was used to pre-optimize the geometry with Dreiding force field method^49^. Finally, the structure of polymer was further accurately optimized by the first-principle method with CP2K package (see details in section “Ab initio molecular dynamics and optimization”)^50^. It’s observed that the final density (1.077 g m^−3^) was very close to the experimental result, and thus illustrating the reliability of our model. All the theoretical simulations in this work were completed in National Supercomputing Center in Shenzhen (NSCS).

### Pore size distribution

Based on the optimized structure, the pore size distribution is calculated by using the Zeo++ software^51^, which can provide the required void accessibility details based on spherical probes and framework atoms of user-specified size. The pore sizes are determined by a spherical probe of radius 1.6 Å (corresponding to N_2_)^26^. Such a probe is considered to be large enough to exclude environments inaccessible to common molecules used as reactants, but small enough to accurately represent the relevant channel corrugations and texture. Furthermore, the maximal diameter for the sphere to freely diffuse in the polymer is calculated by Zeo++, where the default CCDC radii for O, H, C, N are 1.52, 1.09, 1.7, and 1.55 Å, respectively.

### Ab initio molecular dynamics and optimization

The optimization and ab initio molecular dynamics (AIMD) simulation was performed in the mixed Gaussian plane wave scheme using the CP2K code^50,52,53^. The Perdew, Burke, and Ernzrhof (PBE) exchange-correlation functional^54^ was applied and the D3 correction^55^ of Grimme was used to account for the dispersion interactions. The structures were relaxed by using the DZVP-MOLOPT-SR basis set and GTH pseudo potentials^54^. The plane wave cutoff energy and relative cutoff were 650 Ry and 60 Ry, respectively. During the AIMD simulation, sulfate anions, hydronium ions and vanadium ion (with a ratio of 2:2:1), as well as saturated water molecules, were added into the TFCMs to maintain the charge balance. A production run of 10 ps with a time step of 0.5 fs was performed in the NVT ensemble. The simulated temperature was held at 298 K and controlled by the Nosé-Hoover thermostat^56^. The trajectories were recorded every step to analyze the mean square displacement.

### Mean square displacement (MSD)

The mean square displacement (MSD) of proton/vanadium ion was calculated by using

12 $$\frac{1}{N}\mathop {\sum}\limits_{i = 1}^N {\left[ {r_i\left( t \right) - r_i\left( 0 \right)} \right]} ^2$$

where *N* is the total number of proton/vanadium ions in the unit cell, and *r*_i_ *(t)* is the position of the *i*-th ion at time *t*.

## Supplementary information

supplementary information

Supplementary Movie 1

Description of additional supplementary files

## Data Availability

The data that support the findings of this study are available from the corresponding author upon reasonable request. The source data of Fig. 2b–d and Supplementary Figs. 3 and 14 are provided as a Source Data file.

## Source data

source data
