# Publisher Correction: Thin-film composite membrane breaking the trade-off between conductivity and selectivity for a flow battery

**DOI:** 10.1038/s41467-020-16360-z

**Published:** 2020-05-20

**Authors:** Qing Dai, Zhiqiang Liu, Ling Huang, Chao Wang, Yuyue Zhao, Qiang Fu, Anmin Zheng, Huamin Zhang, Xianfeng Li

**Affiliations:** 1 Division of Energy Storage, Dalian National Laboratory for Clean Energy (DNL), Dalian Institute of Chemical Physics, Chinese Academy of Sciences, Dalian, 116023 China; 2 University of Chinese Academy of Sciences, Beijing, 100049 China; 3 State Key Laboratory of Magnetic Resonance and Atomic and Molecular Physics, National Center for Magnetic Resonance in Wuhan, Key Laboratory of Magnetic Resonance in Biological Systems, Wuhan Institute of Physics and Mathematics, Innovation Academy for Precision Measurement Science and Technology, Chinese Academy of Sciences, Wuhan, 430071 China; 4 State Key Laboratory of Catalysis, Dalian Institute of Chemical Physics, Chinese Academy of Sciences, Dalian, 116023 China; 5 School of Materials Science and Engineering, Zhengzhou University, Zhengzhou, Henan 450001 China

**Keywords:** Electrochemistry, Energy, Materials chemistry, Batteries

Correction to: *Nature Communications* 10.1038/s41467-019-13704-2, published online 7 January 2020.

In the original version of this Article, the affiliation details for Qing Dai were incorrectly given as 1 and 3, for Zhiqiang Liu and Ling Huang as 2, for Chao Wang as 3 and 4, Yuyue Zhao as 1 and 3, Anmin Zheng as 2 and 5. This has now been corrected in both the PDF and HTML versions of the Article.

