# Supplementary material for: Thin-film composite membrane breaking the trade-off between conductivity and selectivity for a flow battery
Source: Nat Commun. 2020 Jan 7;11:13. doi: 10.1038/s41467-019-13704-2 (PMC6946707; doi:10.1038/s41467-019-13704-2)
Supplement: Supplementary file 1 — supplementary information [file 41467_2019_13704_MOESM1_ESM.pdf]

# Supplementary Information For

## Thin-film composite membrane breaking the trade-off between conductivity and selectivity for flow battery

by Dai *et al.*

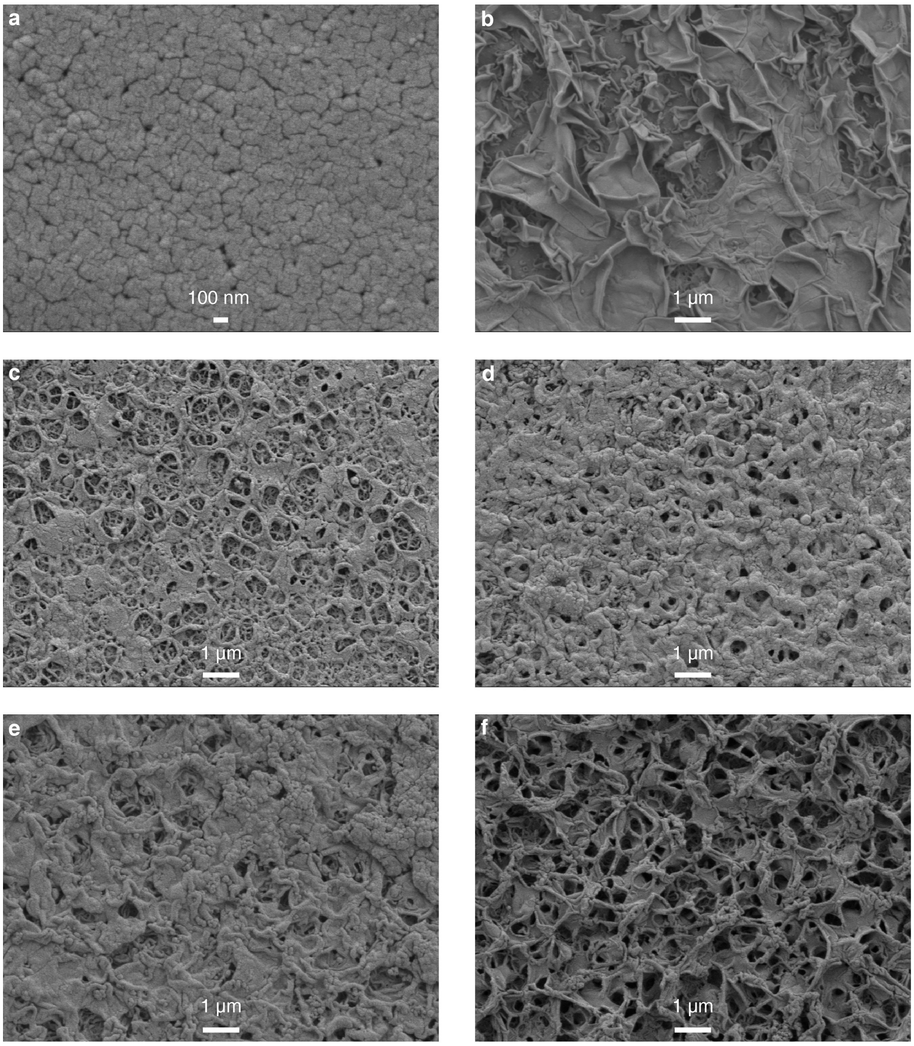


**Supplementary Figure 1. The surface morphologies of thin-film composite membranes with different TMC concentration. a**, Substrate, **b**, IP2-0.02, **c**, IP2-0.15, **d**, IP2-0.3, **e**, IP2-0.4, **f**, IP2-0.8.

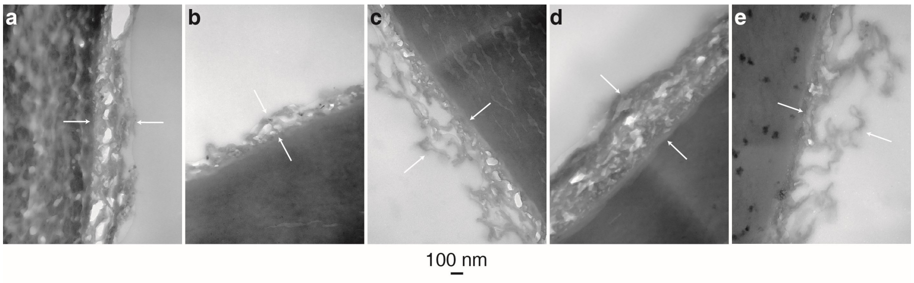


**Supplementary Figure 2. TEM images of membrane cross section.** TEM images of **a**, IP2-0.02 **b**, IP2-0.15 **c**, IP2-0.3 **d**, IP2-0.4 **e**, IP2-0.8. The selective layer is in the region between two arrows.

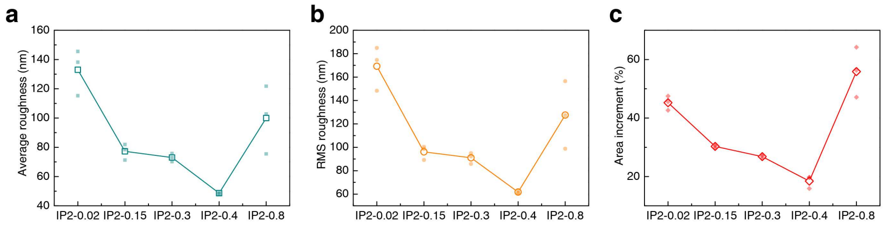


**Supplementary Figure 3. The surface roughness of different TFCMs measured by AFM.** The average roughness (**a**) RMS roughness (**b**) and area increment (the percentage of the surface area higher than the size of the mapping area) (**c**) are the average values of three different 5 × 5 μm mapping areas for each sample. Data behind each average are represented by solid symbols. Source data are provided as a Source Data file.

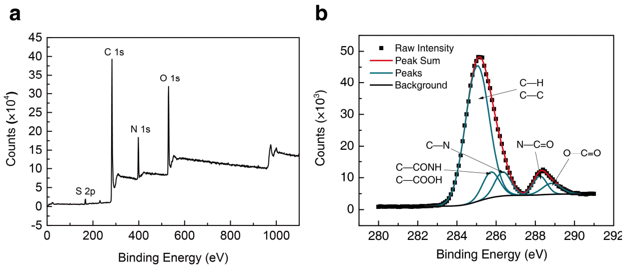


**Supplementary Figure 4. The chemical component of IP2-0.15 detected by XPS. a**, XPS survey spectrum of IP2-0.15. The S 2p signal is attributed to the PES/SPEEK substrate. **b**, High-resolution C1s spectrum of IP2-0.15. IP2-0.15 shows a typical XPS C1s spectrum with C-C, C=C, and C-H at 285.0 eV, β-shift for C-CONH and C-COO at 285.7 eV, C-N at 286.3 eV, N-C=O at 288.2 eV and O-C=O at 288.8 eV. We got these results by referring to the data published by Santanu Karan etc.[1].

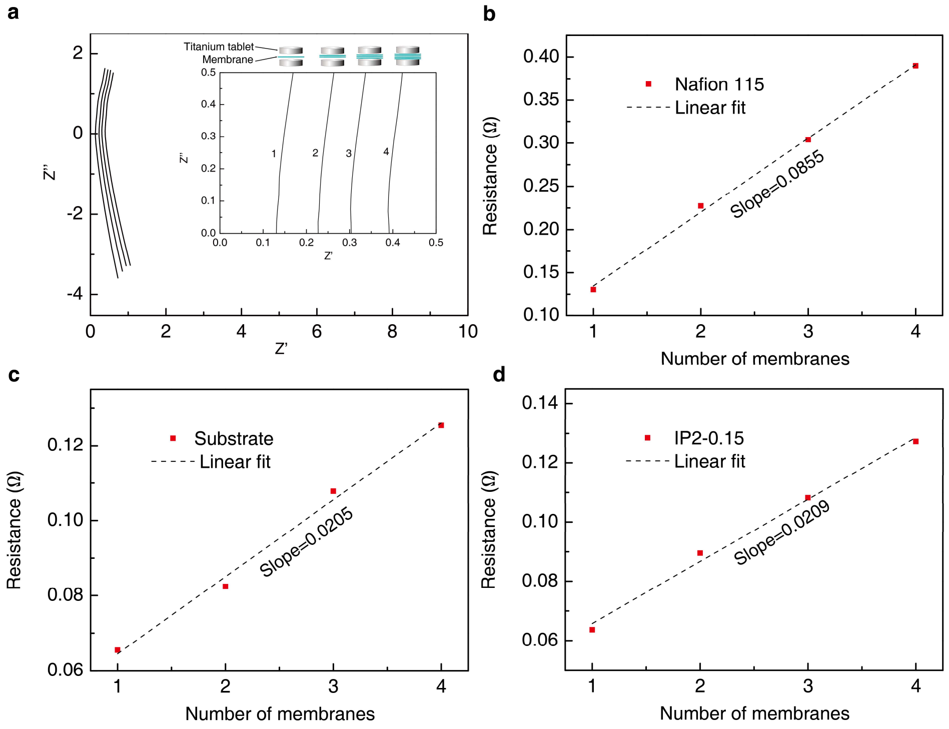


**Supplementary Figure 5. The cross-membrane resistance measured by EIS in 3 M sulfuric acid solution. a**, The EIS plot of different numbers of Nf 115 stacked layer-by-layer. Inset: The magnified EIS plot and the schematic diagram of the testing device. The EIS plot shift to right with the increase of membrane stacked layer-by-layer. The intercept at the real numbers axis is considered as the sum of the total resistance of membranes, the contact resistance and electric resistance of the device. **b**, The change of the resistance with the numbers of Nf 115. The resistance of one membrane was calculated from the slope of the linear fit of the resistance vs. the numbers of membranes stacked layer-by-layer. As a result, the membrane resistance with an effective area of 1.766 $cm^2$ is 0.0855 Ω for this sample. The sum of contact resistance and the electric resistance equal to the intercept of the linear fit line at the vertical axis. The value of the intercept at the vertical axis is 0.0491 Ω which could not be neglected if the membrane resistance is directly measured. The conductivity and area resistance of a membrane is calculated by equation (1) and equation (2), respectively. The effective area is 1.766 $cm^2$ and the thickness of Nafion 115 membrane after swelling in sulfuric acid solution is 138 μm measured with a micrometer caliper. As a result, the conductivity of this Nf 115 sample is 0.091 S $cm^{-1}$ and the area resistance is 0.151 Ω $cm^2$. **c**,**d**, The change of the resistance with the numbers of IP2-0.15 (**c**) and the substrate (**d**). The cross-membrane conductivity of the substrate and IP2-0.15 was measured and calculated with the same procedure as Nf 115.

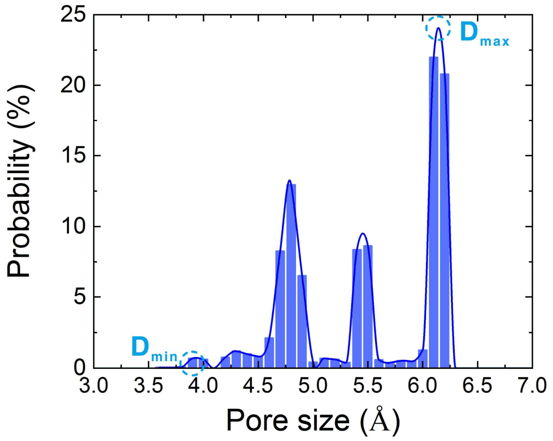


**Supplementary Figure 6.** The pore size distribution of the polyamide framework.

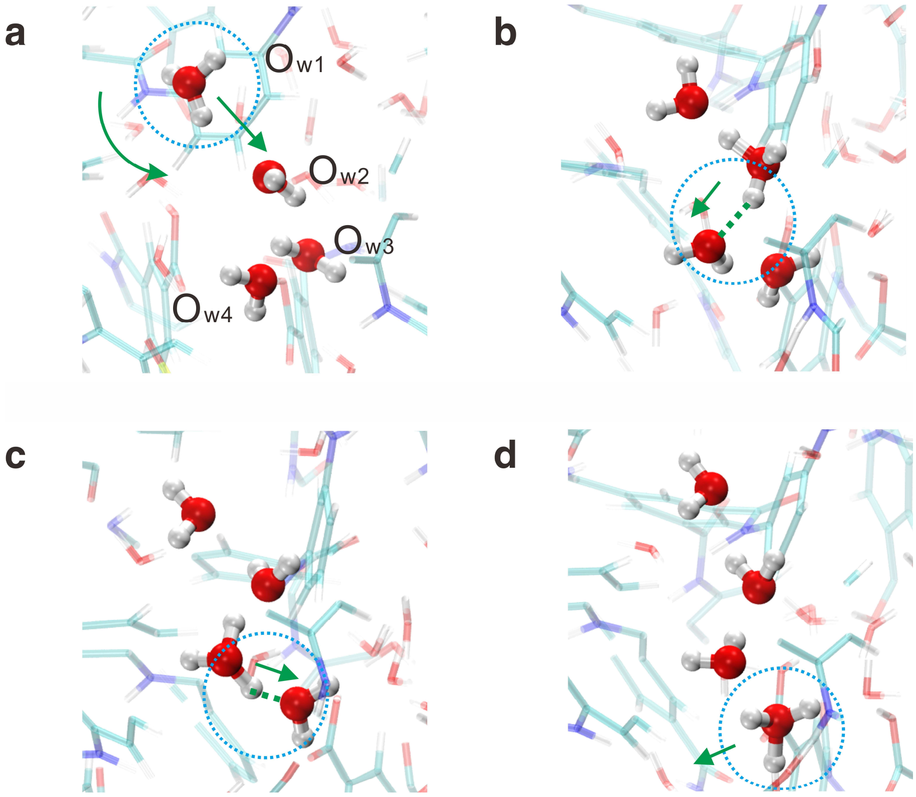


**Supplementary Figure 7.** The snapshots of proton diffusing in TFCMs through water at (a) 0 fs, (b) 250 fs, (c) 325 fs and (d) 375 fs. The red, cyan and white sphere represent O, C and H atom, respectively.

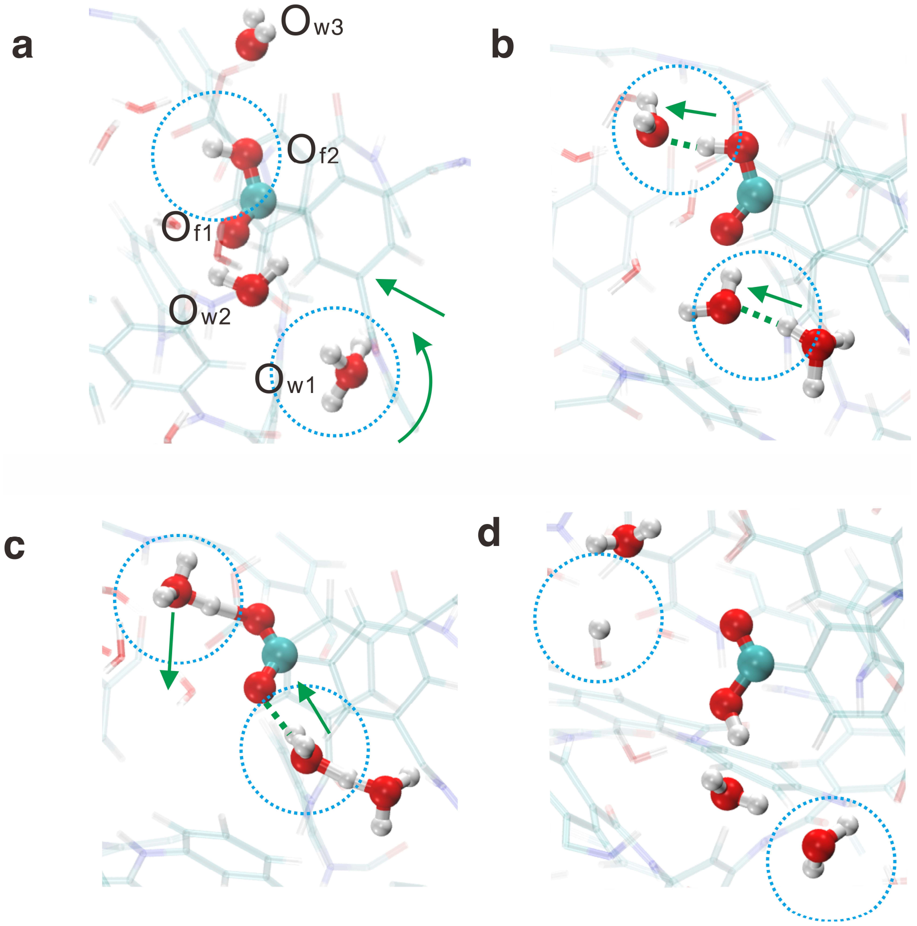


**Supplementary Figure 8.** The snapshots of proton diffusing in TFCMs through water molecules and carboxyl groups at (a) 125 fs, (b) 350 fs, (c) 425 fs and (d) 920 fs. The red, cyan and white sphere represent O, C and H atom, respectively.

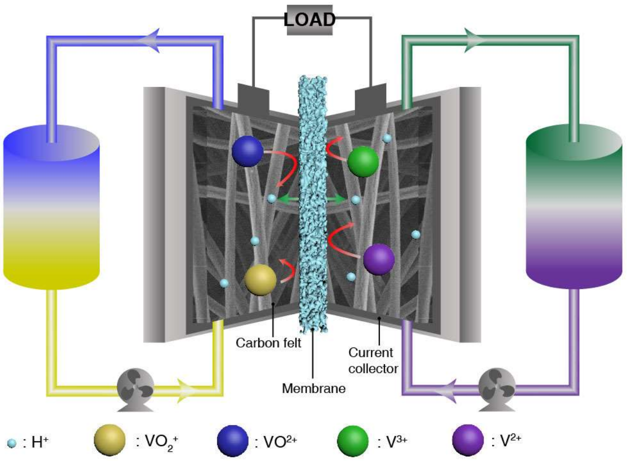


**Supplementary Figure 9. Schematic diagram of the structure of a vanadium single battery.** Electrolytes are pumped into the positive chamber and negative chamber and the vanadium ions react on the carbon felt. The function of the membrane is to resist vanadium ions and conduct protons to complete the internal circuit. The current is collected by graphite carbon felts. Colors represent the color of vanadium ions at different valence states.

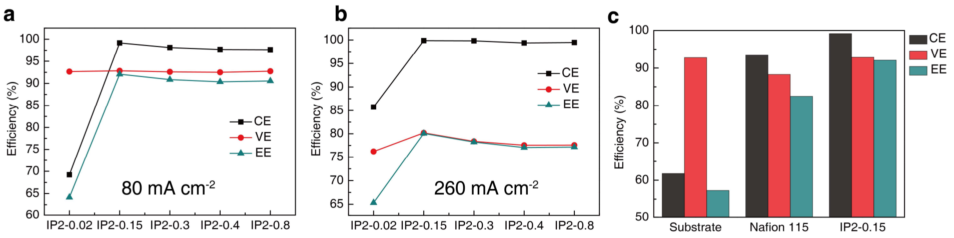


**Supplementary Figure 10. Single battery efficiency of VFBs assembled with different membranes. a**,**b**, Battery performance at current density of 80 mA $cm^{-2}$ (**a**) and 260 mA $cm^{-2}$ (**b**). **c**, Single battery efficiency of VFBs assembled with different membranes at 80 mA $cm^{-2}$. Note that the low VE of IP2-0.02 at 260 mA $cm^{-2}$ was caused by serious electrolyte imbalance.

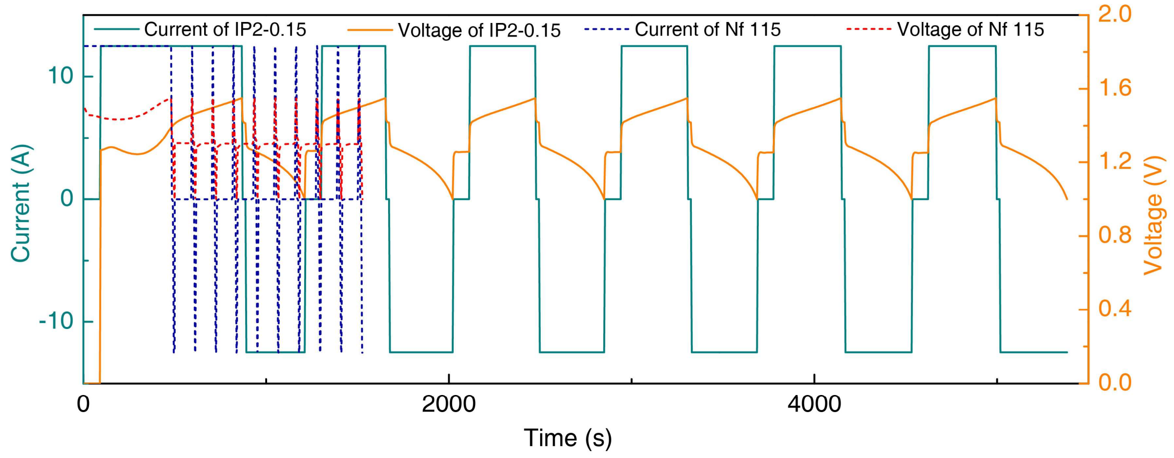


**Supplementary Figure 11. Comparison of charge-discharge curves of IP2-0.15 and Nafion 115 at 260 mA $cm^{-2}$.** With the same volume of electrolyte, the VFB assembled with IP2-0.15 runs one cycle for over 10 min at 260 mA $cm^{-2}$ while the VFB assembled with Nafion 115 finishes one cycle in seconds. This is caused by a large polarization of the VFB assembled with Nafion 115. The voltage curve shows that the voltage increases when charging and decreases when discharging. The voltage declines abruptly at the time when switching from charge to discharge. The decline of the voltage of VFB assembled with Nafion 115 is much larger than that of VFB assembled with IP2-0.15. This result indicates the much larger inner resistance of Nafion 115 than IP2-0.15, and also directly proves that the conductivity of the membrane is the limiting factor of the inner resistance of a VFB. Note that we rest the battery for 90 seconds every one cycle for safety because the charge current is as high as 12.48 A at 260 mA $cm^{-2}$ and the electrode clamps are very hot.

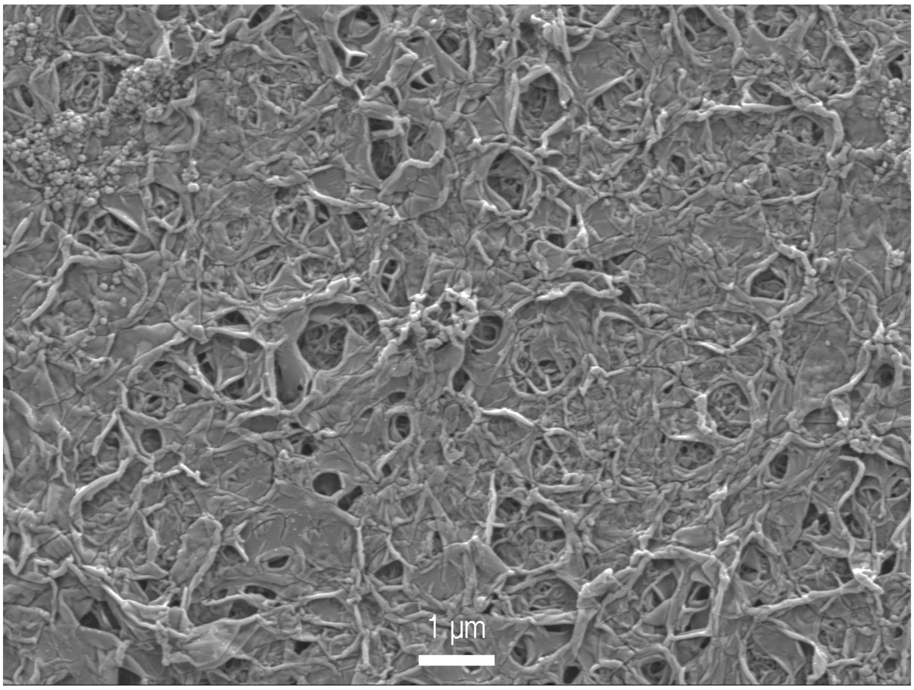


**Supplementary Figure 12. The morphology of IP2-0.15 after 1000 cycles.** The selective layer is well reserved. The selective layer is flatter than before because of the press of electrodes and the hydraulic pressure of electrolyte.

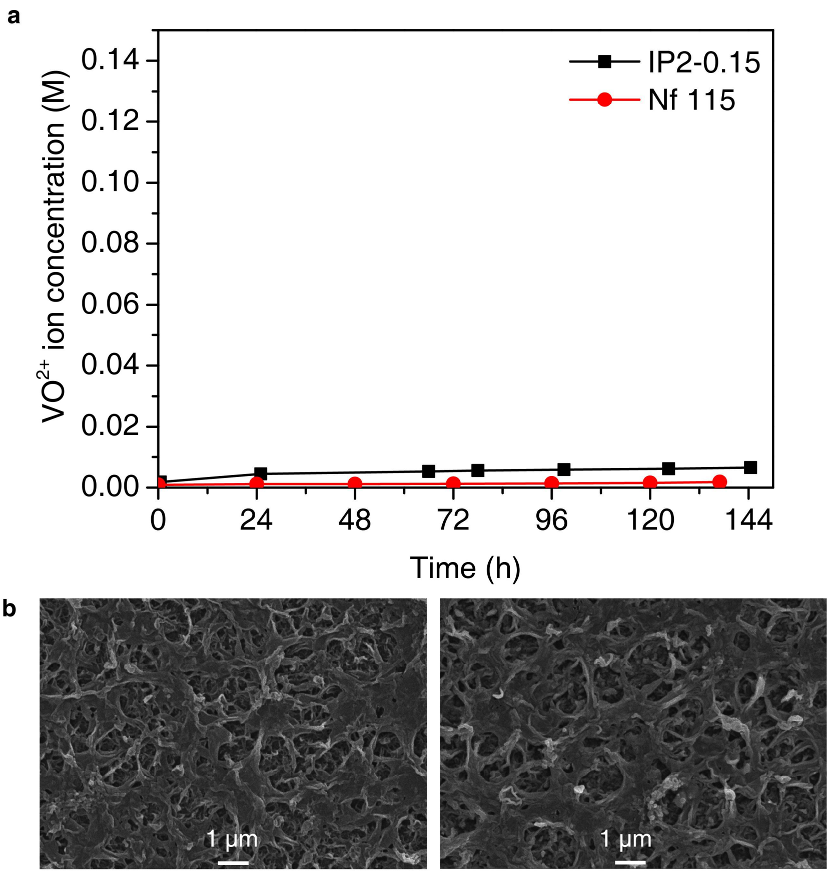


**Supplementary Figure 13. Offline stability test. a,** VO2+ concentration in the soaking solution as a function of time. **b,** The morphology before (left) and after (right) soaking for 7 days.

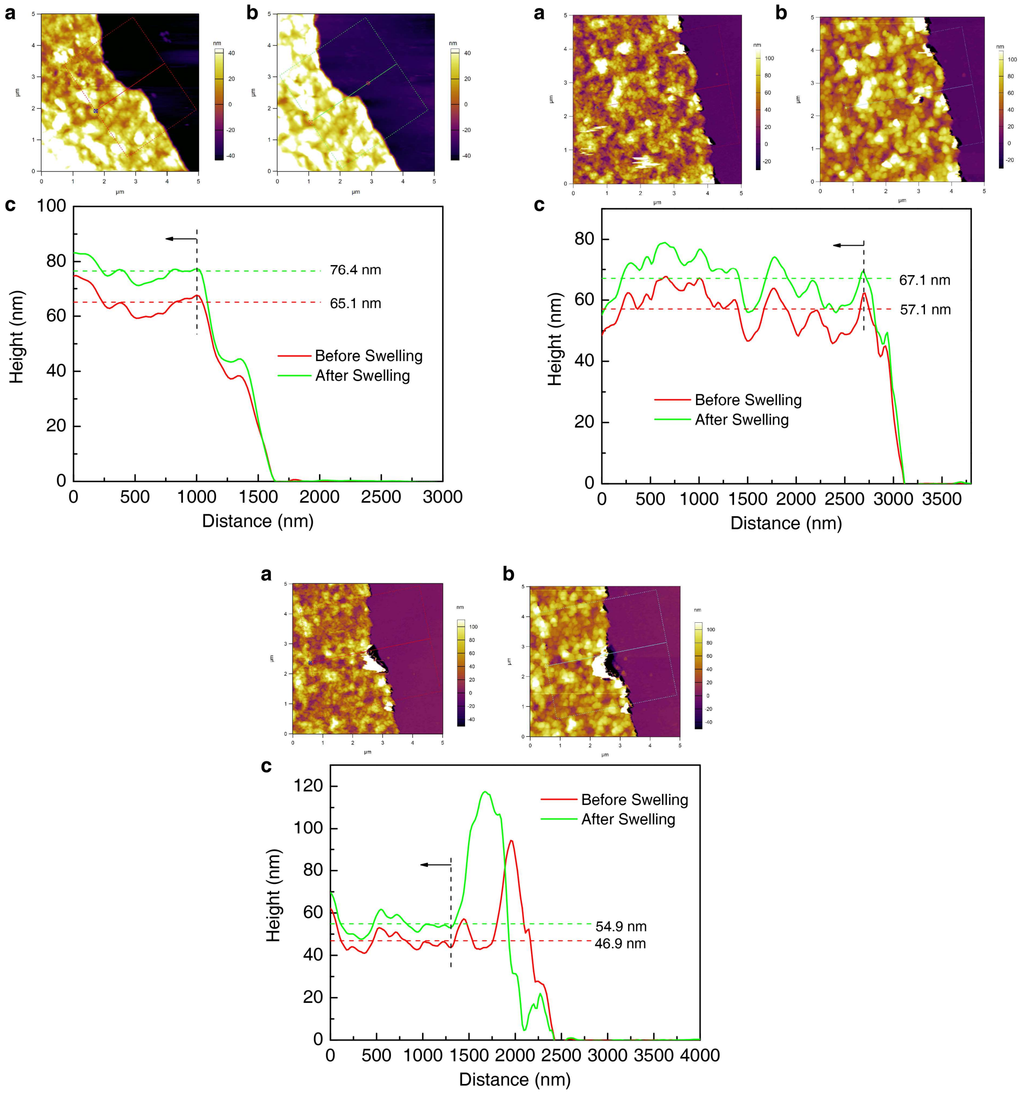


**Supplementary Figure 14. Swelling of the selective layer of IP2-0.15 in water. a**, AFM image before swelling. **b**, AFM image after swelling. **c**, height profile in the selected direction by setting the height of the wafer to 0. The same area was selected for a better comparison. The height at each distance is the mean height in the profile width (4 μm). Data to the left of the black vertical lines are used to determine the thickness. The swelling ratio is 17.3% (average value of 17.4%, 17.1% and 17.5%). Released from the substrate, the height is smaller than the thickness of the selective layer. Source data are provided as a Source Data file.

**Supplementary Table 1. The data to calculate the density of polyamide.**

| $m_1$/g | $m_2$/g | $m_3$/g | $\rho_{true}$/(g cm$^{-3}$) |
|---|---|---|---|
| 24.9164 | 0.2248 | 24.9638 | 1.2672 |
| 24.9180 | 0.1713 | 24.9564 | 1.2889 |
| 24.8438 | 0.1268 | 24.8709 | 1.2718 |
| 24.8438 | 0.1342 | 24.8745 | 1.2966 |
| 24.8438 | 0.1072 | 24.8670 | 1.2762 |

*The density measured by pycnometer is the true density of the sample, which is the density of the part that excludes pores accessible to water molecules. The true density is about 1.28 g cm$^{-3}$. The bulk density after swelling is about 1.09 g cm$^{-3}$ calculated by equation (9). From the previous work[1], the density of polyamide films made in a little higher MPD concentration (3 wt.%, 1.22-1.28 g cm$^{-3}$) is close to the density of our measurement (1.276 g cm$^{-3}$). The density of dry polyamide film measure by Neutron Reflection[2] is between 1.2-1.33 g cm$^{-3}$. Therefore, we believe the result of our measurement is reasonable. Some higher densities were also reported. M.J. Kotelyanskii et al.[3] reports a density of 1.38 g cm$^{-3}$ and S. D. Arthur[4] reports a density within 1.35-1.4 g cm$^{-3}$.

**Supplementary Table 2. The elemental content of the selective layer.**

| | C (%) | N (%) | O (%) | Crosslinking degree[1,5]* |
|---|---|---|---|---|
| XPS data 1† | 75.3 | 9.8 | 14.9 | 0.38 |
| XPS data 2‡ | 69.7 | 11.7 | 18.6 | 0.31 |
| Calculation model | 73.0 | 10.8 | 16.2 | 0.4 |

† Measured by dissolving the substrate and transferring the selective layer onto a silicon wafer.

‡ Measured right on the PES/SPEEK substrate.

*Calculated by equation (10) and (11):

## Supplementary reference


1 Karan, S., Jiang, Z. & Livingston, A. G. Sub–10 nm polyamide nanofilms with ultrafast solvent transport for molecular separation. *Science* **348**, 1347-1351 (2015).

2 Foglia, F. *et al.* Neutron Reflectivity and Performance of Polyamide Nanofilms for Water Desalination. *Adv. Funct. Mater.* **27**, 1701738, (2017).

3 J, K. M., J, W. N. & E, P. M. Atomistic simulation of water and salt transport in the reverse osmosis membrane FT-30. *J. Membr. Sci.* **139**, 1-16 (1998).

4 Structure-property relationship in a thin film composite reverse osmosis membrane. *J. Membr. Sci.* **46**, 243-260 (1989).

5 Akin, O. & Temelli, F. Probing the hydrophobicity of commercial reverse osmosis membranes produced by interfacial polymerization using contact angle, XPS, FTIR, FE-SEM and AFM. *Desalination* **278**, 387-396, (2011).
