# Supplementary figures and images for: Thin-film composite membrane breaking the trade-off between conductivity and selectivity for a flow battery
Source: Nat Commun. 2020 Jan 7;11:13. doi: 10.1038/s41467-019-13704-2 (PMC6946707; doi:10.1038/s41467-019-13704-2)

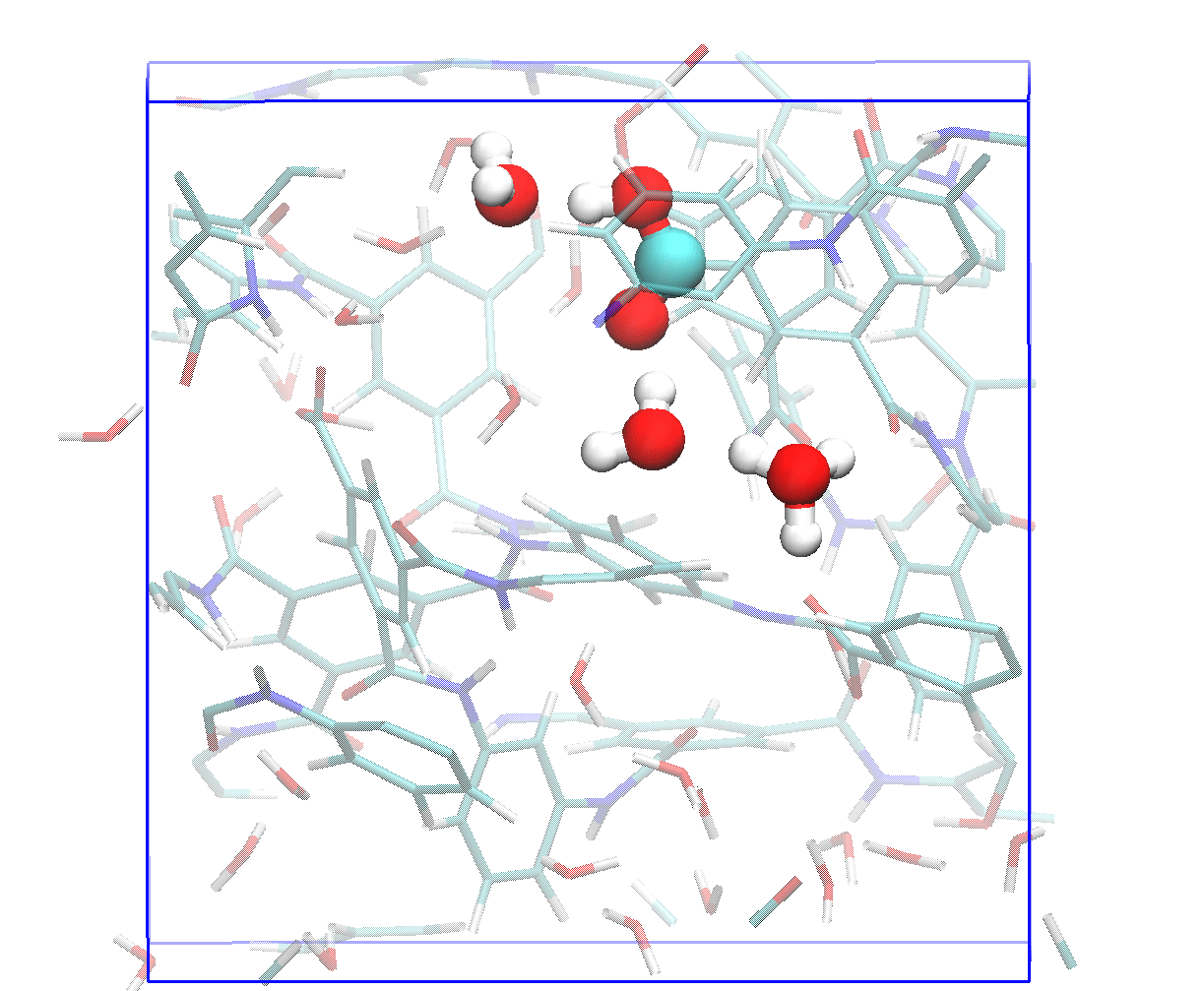

Supplement: Supplementary file 2 — Supplementary Movie 1 [file 41467_2019_13704_MOESM2_ESM.gif]
