# Supplementary material for: Thin-film composite membrane breaking the trade-off between conductivity and selectivity for a flow battery
Source: Nat Commun. 2020 Jan 7;11:13. doi: 10.1038/s41467-019-13704-2 (PMC6946707; doi:10.1038/s41467-019-13704-2)
Supplement: Supplementary file 3 — Description of additional supplementary files [file 41467_2019_13704_MOESM3_ESM.docx]

**Description of Additional Supplementary Files**

**File name: Supplementary Movie 1.**

**Description: The proton transfer in polyamide.** Protons hope between water molecules and carboxyl groups on the polyamide framwork.
